# Comparison of widefield swept-source optical coherence tomography angiography and ultra-widefield fluorescein angiography in the detection of non-perfusion areas in diabetic retinopathy

**DOI:** 10.3389/fendo.2025.1521837

**Published:** 2025-04-08

**Authors:** Chuyun Guo, Ning Xiao, Fang Li, Yue Han, Li Chen, Hongzhuang Chen, Yadan Shen, Xinru Ning, Ruolan Ling, Xi Wang, Lin Zhang, You Wang, Jie Zhong, Jie Li

**Affiliations:** ^1^ Department of Ophthalmology, Sichuan Provincial People’s Hospital, School of Medicine, University of Electronic Science and Technology of China, Chengdu, China; ^2^ Department of Health Management Center & Institute of Health Management, Sichuan Provincial People’s Hospital, University of Electronic Science and Technology of China, Chengdu, China; ^3^ Health Management Medical Center, Chengdu First People’s Hospital, Chengdu, China; ^4^ Sichuan Provincial Key Laboratory for Human Disease Gene Study and Department of Laboratory Medicine, Sichuan Provincial People’s Hospital, School of Medicine, University of Electronic Science and Technology of China, Chengdu, China

**Keywords:** diabetic retinopathy, non-perfusion areas, swept-source widefield optical coherence tomography angiography, ultra-widefield fluorescein angiography, ischemic index

## Abstract

**Purpose:**

To compare the detection of non-perfusion areas (NPAs) in diabetic retinopathy (DR) using 24×20 mm widefield swept-source optical coherence tomography angiography (SS-OCTA) and ultra-widefield fluorescein angiography (UWFA), and to explore NPA distribution patterns.

**Methods:**

This retrospective study included 64 eyes from 48 DR patients who underwent 24×20 mm SS-OCTA and UWFA examinations. NPAs were manually annotated, and the detection rates and distribution patterns across retinal quadrants were analyzed and compared between the two imaging modalities.

**Results:**

Compared to UWFA, the 24×20 mm SS-OCTA scan range missed 53.40% of total NPAs. The detection rates within the SS-OCTA scan range varied across quadrants: 50.58% (superior temporal), 55.33% (inferior temporal), 43.99% (superior nasal), and 43.45% (inferior nasal). NPAs were most prevalent in the inferior nasal region (40.16% of total NPAs). The ischemic index (ISI) derived independently from NPAs identified by the two imaging modalities showed a very strong positive correlation.

**Conclusions:**

Within the scan range of 24×20 mm SS-OCTA, over 50% of total NPAs were missed compared to UWFA. However, OCTA can accurately reveal the degree of retinal ischemia within its field of view. NPA is unevenly distributed in the retina, with the predominant area being the inferior nasal region. This study suggests that this area should be prioritized for assessment in DR management.

## 1 Introduction

Diabetic retinopathy (DR) is the most common microvascular complication of diabetes and is a leading cause of vision impairment and blindness among the working-age population worldwide (1). In DR, prolonged metabolic abnormalities and damage to capillary endothelial cells lead to capillary occlusion, resulting in the formation of non-perfusion areas (NPAs) which enhance retinal ischemia. Several studies have shown that when the area of NPAs reaches a certain threshold, neovascularization (NV) occurs (2, 3). This process marks the transition from non-proliferative diabetic retinopathy (NPDR) to proliferative diabetic retinopathy (PDR). Therefore, NPAs can serve as a potential quantitative biomarker to describe the severity of DR and predict disease progression. These pathological changes are typically not apparent in fundoscopic examination or color fundus photography (CFP) but can be readily observed with fluorescein angiography. Thus, NPAs measurable by fluorescein angiography may provide critical information for monitoring disease progression and assessing the efficacy of treatments aimed at preventing the transition from NPDR to PDR.

Ultra-widefield fluorescein angiography (UWFA) provides a fundus image with an approximately 200°field of view (FOV), covering significantly more of the retina compared to traditional seven-field imaging (4). However, its invasive nature, time-consuming procedure, and potential for dye-induced allergic reactions limit its clinical application. Recently, widefield swept-source optical coherence tomography angiography (SS-OCTA) has emerged as a non-invasive alternative for diagnosing and monitoring DR due to its speed and repeatability. One of the latest developed Toward Pi SS-OCTA system can obtain a 24×20 mm (approximately 120°FOV) retinal blood flow image in a single scan, offering one of the largest retinal detection ranges currently available (5). Numerous studies have demonstrated the feasibility of SS-OCTA in diagnosing DR-related lesions such as microaneurysms (MAs), intraretinal microvascular abnormalities (IRMAs), NPAs, and NV (6, 7). Previous research has found that 24×20 mm SS-OCTA combined with CFP shows excellent concordance with fundus fluorescein angiography (FFA) combined with UWF CFP in grading DR severity, suggesting that SS-OCTA holds promise not only as a diagnostic tool but also for regular monitoring of DR, potentially improving patient outcomes through earlier intervention and more frequent assessments (7–9).

In our clinical practice, we have observed a significant reduction (over 50%) in the use of UWFA examinations for DR patients since the introduction of widefield SS-OCTA. This trend is prevalent in many hospitals. This shift may be attributed to the non-invasive nature of SS-OCTA, its faster acquisition time, and improved patient comfort. While the medical community celebrates the advent of widefield SS-OCTA, we question whether it can truly replace UWFA in managing DR. According to Silva PS et al., 70% of retinal NPAs are located in the mid-peripheral region, a primary target for pan-retinal photocoagulation (10). Previous study also have demonstrated that patients with NPAs predominantly in the peripheral retina are more likely to progress from NPDR to PDR (11). Furthermore, patients with predominantly peripheral NPA distribution are at higher risk of developing diabetic macular edema (DME) (12).

Although the scanning range of commercially available SS-OCTA has gradually expanded, there may still be issues with lesion detection compared to UWFA. Therefore, this study aims to investigate the differences in NPAs detection between 24×20 mm SS-OCTA and UWFA in patients with DR, and the implications of these differences. The findings of this study will be critical in determining whether SS-OCTA can serve as a comprehensive, non-invasive alternative to UWFA in detecting NPAs and guiding treatment decisions in DR management.

## 2 Materials and methods

### 2.1 Participants

This cross-sectional retrospective study included diabetic patients who underwent both UWFA and SS-OCTA in the Ophthalmology Department of Sichuan Provincial People’s Hospital between December 2022 and September 2024. The study was approved by the Ethics Committee of Sichuan Provincial People’s Hospital, and all procedures were conducted in accordance with the principles of the Declaration of Helsinki.

All enrolled patients underwent a comprehensive ophthalmic evaluation before the UWFA examination. This evaluation included best-corrected visual acuity (BCVA), intraocular pressure (IOP) measurement, slit-lamp examination of the anterior segment, and slit-lamp fundus examination. The interval between the UWFA and SS-OCTA examinations did not exceed one month, and no other ophthalmic treatments were administered during this period.

### 2.2 Inclusion and exclusion criteria

Inclusion Criteria: Diabetic patients who underwent both UWFA and SS-OCTA within one month.

Exclusion Criteria: Patients with prior DR treatments (e.g., laser photocoagulation, anti-VEGF injections, vitrectomy), other ocular conditions (e.g., retinal or choroidal diseases, glaucoma), suboptimal imaging quality, or incomplete imaging data were excluded (e.g., media opacity affecting image interpretation, lack of NPAs in UWFA and SS-OCTA images, or leakage in UWFA images affecting NPA annotation.

### 2.3 Standard operating procedure

#### 2.3.1 Definition and annotation of NPAs

NPAs were defined as regions of capillary dropout equal to or greater than one-quarter of the optic disc area, following previously established criteria in DR research (7).

#### 2.3.2 Image quality assessment Standards

The image quality assessment for SS-OCTA is first conducted using system-fixed parameters to score signal strength, excluding images with scores lower than 7. Additionally, images with NPAs annotations hindered by eyelash occlusion or artifacts are excluded. The image quality assessment for UWFA is initially carried out by two annotators and one retinal vitreous disease expert. Since UWFA consists of multiple time-point images (i.e., dozens of photographs for each eye), we enhanced NP detection by adjusting the angiographic sequence timing.Images selected for this study are those with complete contrast, proper eye positioning, and minimal occlusion from eyelashes or eyelids, maximizing retinal visibility.

#### 2.3.3 Annotation process

Annotators: Two masked ophthalmologists independently annotated all UWFA and 24×20 mm SS-OCTA images after receiving standardized annotation training.

Image Acquisition Method: The images were acquired using the 24×20 mm SS-OCTA with the Tianpai BM-400K (BM-400K BMizar, TowardPi Medical Technology, Beijing, China), which has a lateral resolution of 10 µm and an axial optical resolution of 3.8 µm. The A-scan depth of this device is 6.0 mm. The 24×20 mm OCTA consists of 1536 A-scans and 1280 B-scans. UWFA was performed using the Optos P200DTx (Optos PLC, Dunfermline, United Kingdom), with retinal images captured 6-7 seconds after the intravenous injection of the contrast agent.

Standardized Protocol: During the image acquisition process, all participants underwent non-dilated imaging. The imaging parameters were kept consistent to ensure that each image was acquired under the same conditions, minimizing the impact of external factors on the results. All UWFA images were acquired by the same operator, and all SS-OCTA images were also obtained by the same operator, using the same equipment and standardized operating techniques, thereby reducing the potential influence of operator bias on the results.

Annotation Tool: The annotation of NP was performed using the built-in software of the respective devices. The accuracy of NP annotation between the two devices has been validated (13, 14), and the annotations between the two image types were not cross-referenced.

Annotation Consistency: The annotation was performed independently by two ophthalmologists without cross-referencing their results. Any discrepancies in the annotations were resolved by a senior retinal specialist. The final NP annotation map was obtained after resolving the discrepancies. Consistency Evaluation: The consistency between the annotators was assessed using intraclass correlation coefficients (ICC). The ICC was 0.979 for SS-OCTA and 0.918 for UWFA, indicating excellent consistency.

#### 2.3.4 Image partitioning and visible area calculation

Image Partitioning: The images were divided into four quadrants by drawing vertical and horizontal lines through the foveal center, creating the following quadrants: superior temporal, inferior temporal, superior nasal, and inferior nasal.

Visible Area Calculation: The visible area in each quadrant was calculated by annotation, excluding areas obscured by the eyelid, eyelashes, or peripheral regions with unclear visibility.

#### 2.3.5 Image overlay alignment

For left-eye images, they were horizontally flipped to maintain consistency. The unannotated 24×20 mm SS-OCTA images were overlaid onto the final annotated UWFA images, aligned based on the optic disc, macula, and major blood vessels (15). This alignment process helped determine the positioning of the 20×24 mm SS-OCTA scan range within the UWFA images.

#### 2.3.6 Grid overlay alignment

A 45×85 mm semi-transparent grid was overlaid onto the aligned images for coordinate calculation in the heatmap. The macula and optic disc were uniformly aligned across all images to ensure consistency in the coordinate calculation standard.

#### 2.3.7 NPA frequency calculation and heatmap generation

The NPA frequency for each patient’s semi-transparent grid was calculated, with areas containing NP marked as 1 and areas without NP marked as 0. The frequency of NP occurrence was calculated across 64 eyes. Based on these data, a heatmap of NP distribution was generated to illustrate the distribution of NP in various regions (see **Supplementary Figure 1**).

### 2.4 Evaluation of image annotation difficulty

To assess annotation difficulty, UWFA and corresponding SS-OCTA images from 5 randomly selected patients were rated by 5 masked ophthalmology residents on a scale from 0 (very difficult) to 10 (very easy) based on the perceived difficulty of annotating NPAs.

### 2.5 Data analysis

Statistical analysis was performed using Statistical Package for the Social Sciences version 26.0 (SPSS, IBM, Armonk). Data are presented as mean ± standard deviation (SD). The consistency between ischemic index (ISI) values obtained from SS-OCTA and UWFA was assessed using regression curve estimation, with the degree of consistency expressed by Spearman’s Rank Correlation Coefficient (ρ), ρ>0.9 means a perfect positive correlation, 0.7< ρ < 0.9 means a very strong positive correlation, 0.5 < ρ < 0.7 means strong positive correlation, 0.3 < ρ < 0.5 means a moderate positive correlation, and ρ< 0.3 means a weak correlation. Scatter plots were generated using Origin version 9.1 (Origin, OriginLab, Northampton). Heatmaps were created using Python (Python, Python Software Foundation, Beaverton). ICC was used to evaluate consistency between two masked ophthalmology researchers, with ICC values <0.5 indicating poor, 0.5–0.75 moderate, 0.75–0.9 good, and >0.9 excellent consistency.

## 3 Results

### 3.1 Demographics

A total of 113 diabetic patients (172 eyes) who underwent both UWFA and SS-OCTA examinations at the Ophthalmology Department of Sichuan Provincial People’s Hospital between December 2022 and September 2024 were initially screened for eligibility. Of these, 48 participants (64 eyes) met the inclusion criteria and were included in the final analysis. The mean age of the participants was 52.00 ± 8.23 years. The duration of diabetes since diagnosis ranged from 2 to 23 years, with a mean duration of 10.59 ± 5.75 years. Among the included eyes, 31 were diagnosed with PDR, 21 with severe NPDR, and 12 with mild to moderate NPDR (**Table 1**).

**Table 1:** Demographic characteristics of patients included in the analysis.

Parameters	Date
Mean age (year)	52.00 ± 8.23
Gender ration (F/M)	19/29
Type of diabetes (n=patients)	
Type 1	0
Type 2	48
Duration of Diabetes (years)	10.59 ± 5.75
DR clinical severity grade (n=eyes)	64
Mild and Moderate NPDR	12
Severe NPDR	21
PDR	31

DR, diabetic retinopathy; NPDR, non-proliferative diabetic retinopathy; PDR, proliferative diabetic retinopathy.

### 3.2 Distribution of NPAs in DR patients

**Figure 1:**
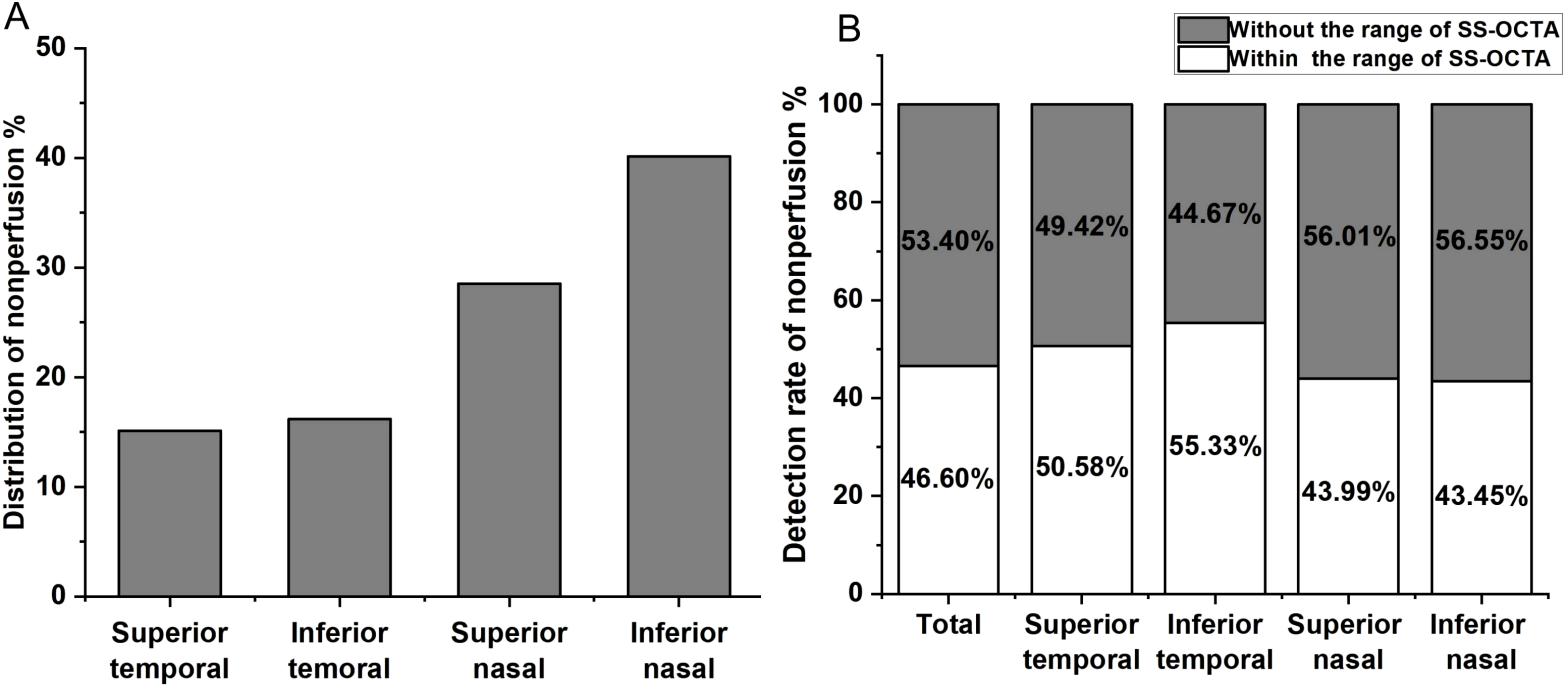
**(A)** Distribution of non-perfusion areas (NPAs) across the four quadrants of the retina. The percentage of NPAs in the superior temporal quadrant is 15.13%, inferior temporal quadrant 16.17%, superior nasal quadrant 28.54%, and inferior nasal quadrant 40.16%. **(B)** Detection of NPAs within the scanning range of SS-OCTA in different retinal quadrants. The overall undetected NPAs rate is 53.40%, with 49.42% in the superior temporal quadrant, 44.67% in the inferior temporal quadrant, 56.55% in the superior nasal quadrant, and 56.55% in the inferior nasal quadrant.

**Figure 2:**
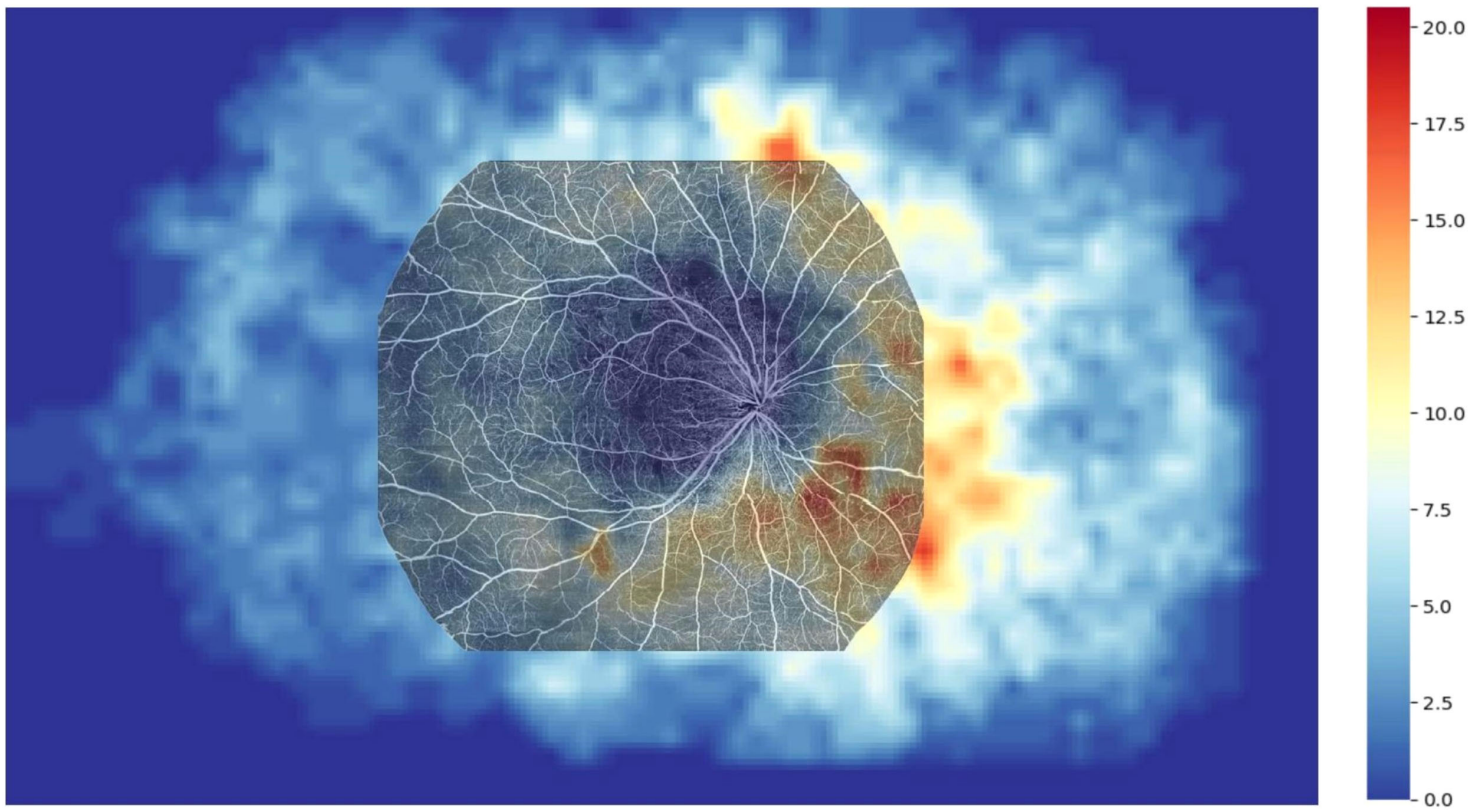
Heatmap illustrating the distribution of NPAs in the ultra-widefield angiography (UWFA) image, overlaid with a 20×24 mm OCTA image. The overlay reveals that 53.40% of NPAs are missed in the scanning range of 20×24 mm SS-OCTA examination.

Using the UWFA images overlaid onto a customized 45×85 grid, we identified a total of 15,422 NPA occurrences. The distribution of NPAs was as follows: the superior temporal quadrant accounted for 2,333 NPAs (15.13% of the total), the inferior temporal quadrant for 2,494 NPAs (16.17%), the superior nasal quadrant for 4,401 NPAs (28.54%), and the inferior nasal quadrant for 6,194 NPAs (40.16%) (**Figure 1A**). A heatmap was generated based on the frequency of NPA occurrences, with SS-OCTA images overlaid onto the heatmap, aligned with corresponding scan range coordinates (**Figure 2**).

### 3.3 Different ages and disease duration on the distribution of NP

**Figure 3:**
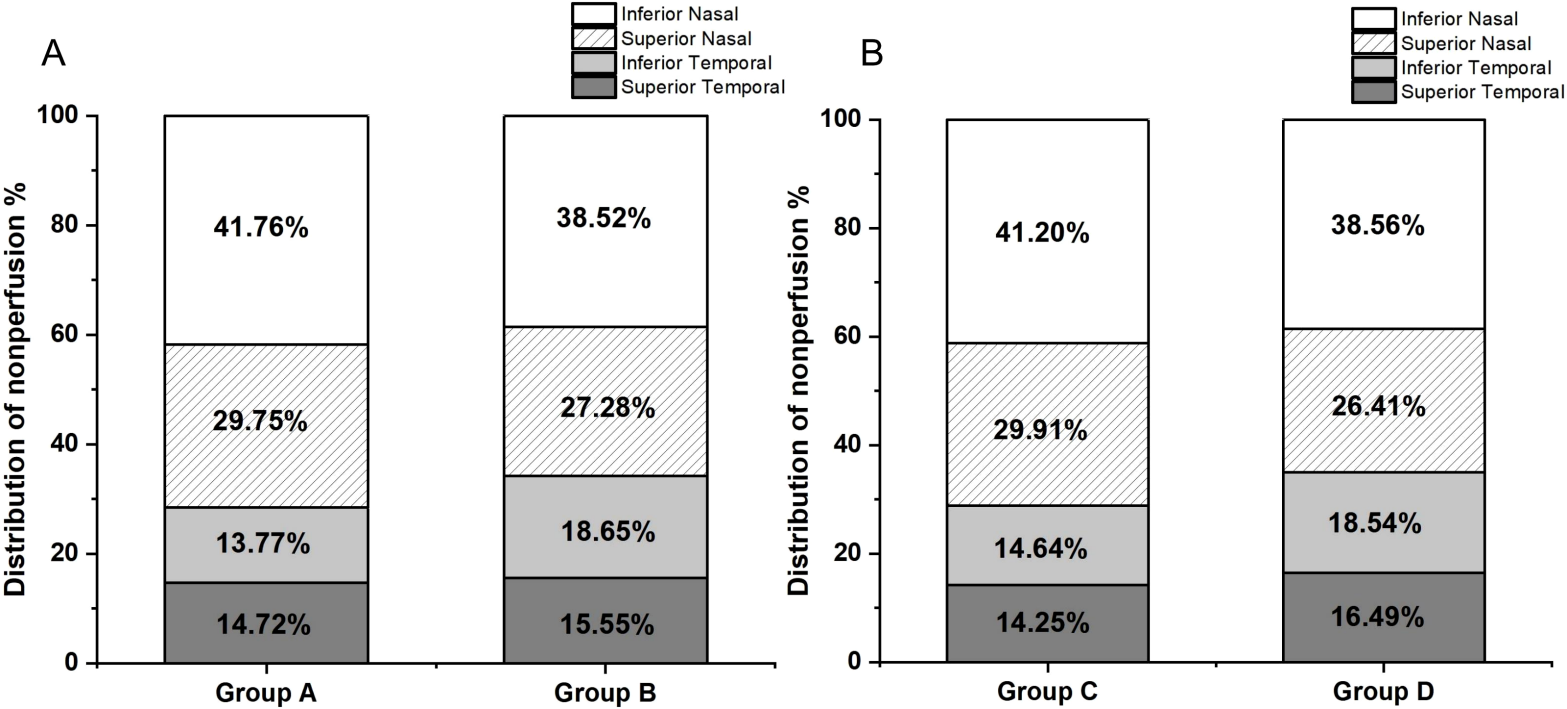
**(A)** Group A (aged 36-52 years, 33 participants) had NP distribution as follows: superior temporal 14.72%, inferior temporal 13.77%, superior nasal 29.75%, and inferior nasal 41.76%. Group B (aged 53-69 years, 31 participants) had NP distribution as follows: superior temporal 15.55%, inferior temporal 18.65%, superior nasal 27.28%, and inferior nasal 38.52%. **(B)** Group C (disease duration 2-10 years, 35 participants) had NP distribution as follows: superior temporal 14.25%, inferior temporal 14.64%, superior nasal 29.91%, and inferior nasal 41.20%. Group D (disease duration 11-23 years, 29 participants) had NP distribution as follows: superior temporal 16.49%, inferior temporal 18.54%, superior nasal 26.41%, and inferior nasal 38.56%.

Since the patients’ ages followed a normal distribution, the average age of 52 years was used as the stratification point, dividing the patients into two groups: 36-52 years (Group A) and 53-69 years (Group B). Group A included 5 eyes with mild to moderate NPDR, 11 eyes with severe NPDR, and 17 eyes with PDR, while Group B included 7 eyes with mild to moderate NPDR, 10 eyes with severe NPDR, and 14 eyes with PDR. The distribution of NP in both groups was illustrated using bar charts (**Figure 3A**). Similarly, since the disease duration did not follow a normal distribution, the median disease duration of 10 years was used as the stratification point, dividing the patients into two groups: 2-10 years (Group C) and 11-23 years (Group D). Group C included 5 eyes with mild to moderate NPDR, 11 eyes with severe NPDR, and 19 eyes with PDR, while Group D included 0 eyes with mild to moderate NPDR, 10 eyes with severe NPDR, and 12 eyes with PDR. The distribution of NP in both groups was also represented using bar charts (**Figure 3B**). The results of the NP distribution pattern showed that, except for Group A, where the inferior temporal quadrant was less than the superior temporal quadrant, in Groups B, C, and D, the distribution followed the pattern: superior temporal quadrant < inferior temporal quadrant < superior nasal quadrant < inferior nasal quadrant.

### 3.4 Comparison of NPA detection rates between overall UWFA and UWFA within the 24×20 mm OCTA scanning area

We aligned SS-OCTA images to a heatmap based on fixed anatomical landmarks, including the optic disc, macular center, and vascular patterns, to compare the detection rates of NPAs within the 24×20 mm SS-OCTA scan area. UWFA-labeled NPAs were used as the gold standard. In the UWFA images, a total of 15,422 NPAs were identified, of which 7,187 (46.60%) were located within the SS-OCTA scan range. This indicates that 53.40% of the NPAs were outside the SS-OCTA scanning area. Quadrant-specific detection rates within the SS-OCTA range were as follows: 50.58% in the superior temporal quadrant, 55.33% in the inferior temporal quadrant, 43.99% in the superior nasal quadrant, and 43.45% in the inferior nasal quadrant (**Figure 1B**).

### 3.5 Comparison of ischemic index detected by UWFA and 24×20 mm SS-OCTA

**Figure 4:**
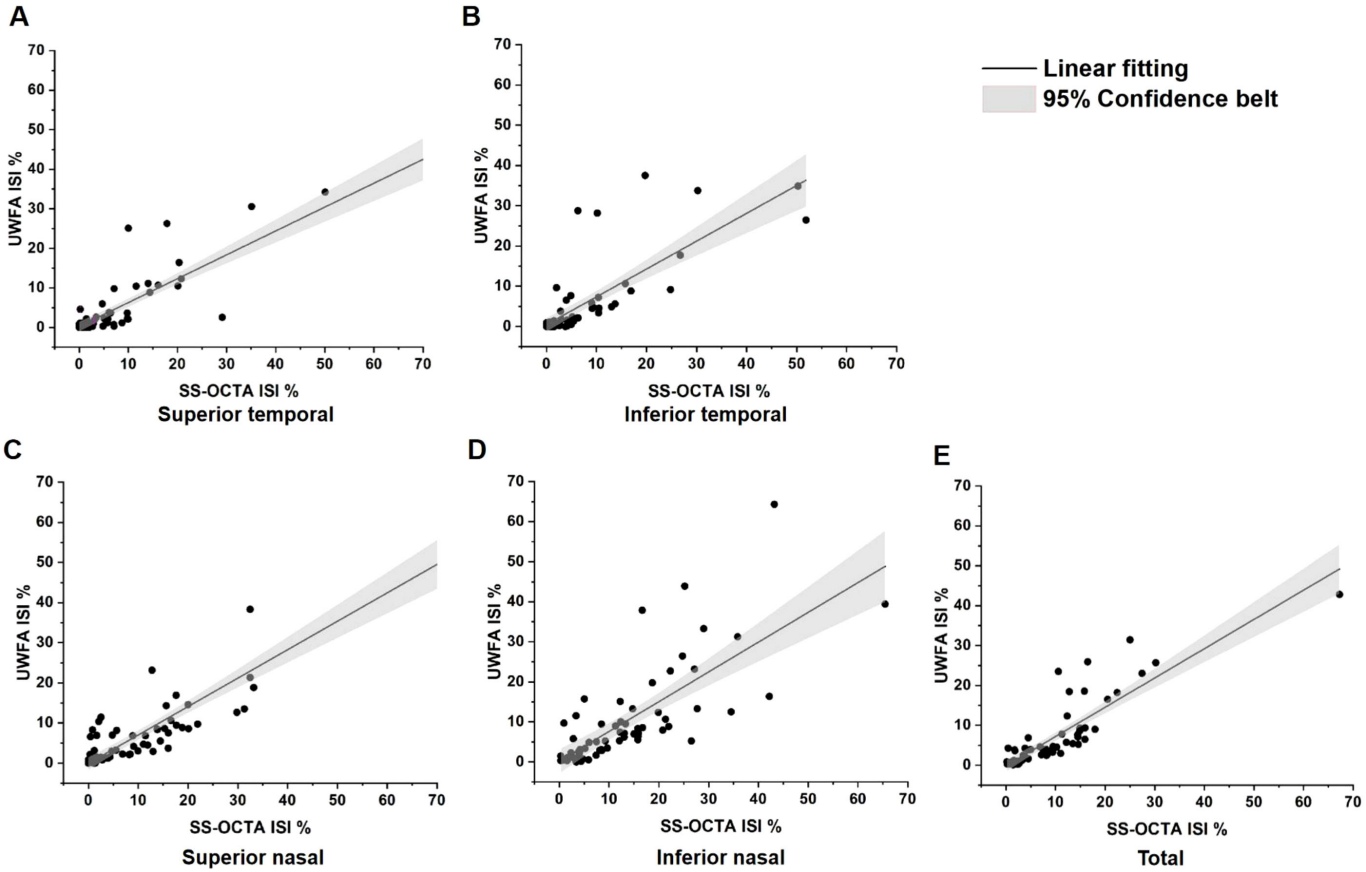
Linear correlation analysis between ischemic index (ISI) values of SS-OCTA and UWFA. NPAs and visible areas were annotated using the build-in software of each imaging device. **(A)** Total SS-OCTA vs. total UWFA ISI, ρ=0.88, p<0.001; **(B)** Superior temporal ISI comparison, ρ=0.81, p<0.001; **(C)** Inferior temporal ISI comparison,ρ=0.83, p<0.001; **(D)** Superior nasal ISI comparison, ρ=0.79, p<0.001; **(E)** Inferior nasal ISI comparison, ρ=0.79, p<0.001. These results demonstrate a very strong positive correlation between the ISIs of SS-OCTA and UWFA. ρ, Spearman’s Rank Correlation Coefficient.

ISI was calculated as the ratio of the NPA to the visible retinal area. For UWFA, the overall ISI was 6.85 ± 8.60%, 95% CI (4.70%, 9.00%), with quadrant-specific values of 4.79 ± 8.92%, 95% CI (2.56%, 7.02%) in the superior temporal quadrant, 5.09 ± 9.32%, 95% CI (2.76%, 7.42%) in the inferior temporal quadrant, 7.10 ± 9.86%, 95% CI (4.64%, 9.56%) in the superior nasal quadrant, and 10.32 ± 12.24%, 95% CI (7.26%, 13.38%) in the inferior nasal quadrant. For SS-OCTA, the overall ISI was higher at 9.52 ± 10.19%, 95% CI (6.98%, 12.07%) with quadrant-specific values of 7.51 ± 12.95%, 95% CI (4.28%, 10.75%), 6.69 ± 10.49%, 95% CI (4.07%, 9.31%), 10.11 ± 12.14%, 95% CI (7.08%, 13.14%) and 13.64 ± 12.30%, 95% CI (10.57%, 16.71%). The Spearman’s Rank Correlation Coefficient (ρ) values between the ISI obtained from UWFA and SS-OCTA in the total retina and the four quadrants were 0.88, 0.81, 0.83, 0.79 and 0.79, respectively, indicating a very strong positive correlation (**Figure 4**).

### 3.6 Comparison of NPA detection difficulty between UWFA and 20×24 mm SS-OCTA images

**Figure 5:**
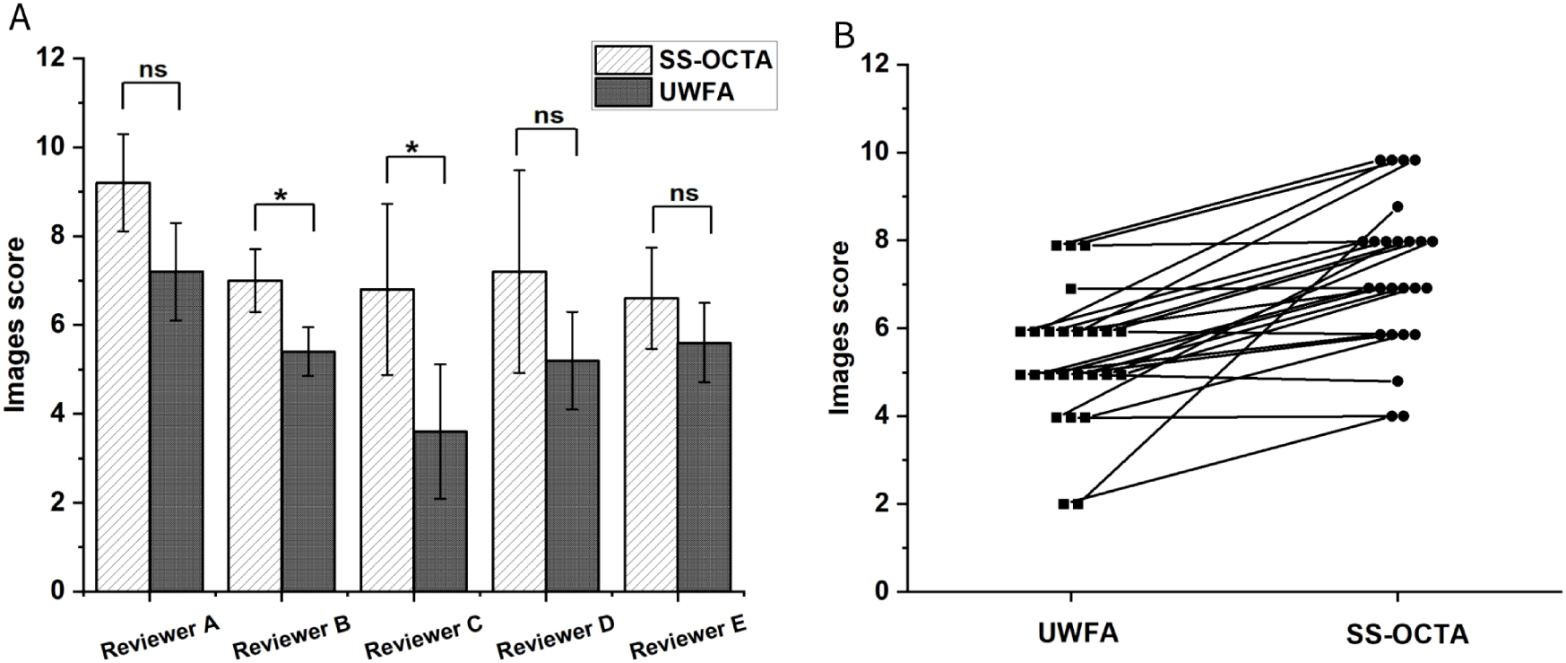
**(A)** Mean difficulty scores for NPA annotations assigned by five researchers across two different imaging results from five randomly selected patients. The comparison assesses whether there is a statistically significant difference in the scores given by the same researcher for the two types of imaging results. P-value of less than was considered statistically significant. **(B)** Paired line plot comparing two imaging results from the same patient. In all cases, the SS-OCTA score is greater than or equal to the UWFA score. The Wilcoxon test indicates a statistically significant difference with a p-value of less than 0.001. ns means no significance, * means p<0.05.

Five ophthalmology residents annotated NPAs on five randomly selected UWFA images and corresponding SS-OCTA images. The UWFA images had an average difficulty score of 5.4 ± 0.94, while SS-OCTA images scored 7.36 ± 1.16 (**Figure 5A**). SS-OCTA consistently received higher or equal scores across all evaluators. A paired Wilcoxon test showed a statistically significant difference in difficulty scores between the two modalities (p <0.001) (**Figure 5B**), indicating that SS-OCTA images presented greater challenges in NPA identification for the residents.

## 4 Discussion

In this retrospective comparative study, we examined the differences in NPA detection between overall UWFA and local UWFA in 24×20 mm SS-OCTA scan area in patients with DR. Our findings indicate that the NPA detection rate of SS-OCTA’s scan area was significantly lower, with an overall loss rate of 53.40%. This suggests that 24×20 mm SS-OCTA can not fully replace UWFA for the diagnosis of NPAs in DR patients. Despite this limitation, there was a very strong positive correlation between the ISI obtained from both imaging modalities, underscoring the utility of SS-OCTA in providing relevant ischemic information. We also analyzed the distribution of NPAs across the four retinal quadrants and found that NPAs were most frequently located in the inferonasal quadrant. Regarding the ease of NPA detection, our assessment indicates that SS-OCTA outperforms UWFA in delineating and interpreting NPA boundaries within its scanning range. This suggests that SS-OCTA provides clearer images for evaluating the extent of retinal ischemia.

To calculate the detection rate of NPAs, we identified the SS-OCTA scan area within the UWFA images and compared NPAs detected within the visible areas of both modalities. Previous studies, including ours and that of Cui et al., have demonstrated the utility of SS-OCTA in diagnosing DR (7, 8), with higher detection rates for NPAs and NV within its scan range (8, 15, 16). However, this study suggests that even the widest commercially available SS-OCTA field of view cannot match the comprehensive coverage of UWFA. Our findings show that SS-OCTA has an NPA detection loss rate exceeding 50%, particularly missing NPAs in the mid-peripheral retina, a region closely associated with DR progression. These findings are consistent with those of Ashraf M., who highlighted the value of UWFA in assessing NPAs for more accurate evaluation of DR severity and progression risk (17). The limitations of traditional ETDRS 7-field imaging in detecting predominantly peripheral lesions (PPL) further emphasize the necessity of widefield imaging (18). The restricted FOV in SS-OCTA, while effective for central ischemia detection, may lead to the omission of clinically significant NPAs, potentially resulting in underestimation of disease severity and suboptimal treatment decisions.

The distribution of NPAs in DR patients in our study aligns with previous research, showing peripheral NPAs are more common than those in the posterior pole. We used a quadrant-based analysis centered on the macula to standardize comparisons. Our findings revealed the inferonasal quadrant consistently had the largest NPA area, highest frequency, and most severe ischemia. This pattern is consistent with Tomita et al. (19), who noted higher blood flow in the upper retina, making the lower retina more susceptible to ischemia. The nasal region’s vascular structure, with fewer anastomoses between superior and inferior vessels, may also explain its higher NPA incidence. Minor discrepancies in NPA measurements across methods could result from image acquisition issues, such as obstructions by eyelashes or eyelids, reducing visible area, particularly in the inferior temporal quadrant.

The asymmetric distribution of NPAs, with a pattern of inferior nasal > superior nasal > superior temporal > inferior temporal NPAs, is a compelling topic. Previous studies by Khoobehi (20) and Guduru (21) demonstrated that as DR severity increases, oxygen saturation in retinal arterioles and venules also rises. This increase is attributed to blood redistribution, where retinal circulation bypasses NPAs, leading to reduced oxygen utilization in these areas and consequently higher oxygen saturation in the surrounding vasculature. Enrico used 12×12 OCTA to observe whether the perfusion density in healthy eyes shows unevenness, and found that the macular region has the highest perfusion density, while the perfusion density in the temporal region is lower than that in the superior and inferior areas (22). This suggests that in healthy eyes, the temporal region has lower deep capillary plexuses perfusion, making it more prone to capillary loss. Therefore, we speculate that in the early stages of DR, the retinal temporal region in affected individuals already exhibits lower perfusion density. Hafner J’s study on type 2 diabetic patients without DR revealed a gradient of retinal oxygen saturation, with the highest levels in the superior nasal quadrant, followed by the inferior nasal, superior temporal, and inferior temporal quadrants (23). This raises the question of whether, at the onset of DR, the superior nasal and inferior nasal regions are more prone to severe pathological changes compared to the temporal quadrants. The distribution pattern observed in our study closely mirrors this sequence, suggesting that retinal ischemia is not uniformly distributed across the retina. These findings underscore the importance of focusing on the inferonasal region during early DR screening and progression monitoring, as this region appears to be the most susceptible to early ischemic damage.

Our study also found that SS-OCTA has a 53.40% NPA detection loss rate compared to UWFA, primarily in the mid-peripheral retina beyond the 24×20 mm scan area. Additionally, in some regions within the 24×20 mm range, UWFA detected blood flow whereas SS-OCTA identified no capillary perfusion. This discrepancy likely stems from SS-OCTA’s limitation in detecting vessels with low blood flow velocity (16).

A total of 113 patients (172 eyes) were initially included in this study, with 52.33% of exclusions solely due to poor OCTA image quality or image artifacts. These findings highlight the critical importance of image quality in the accurate assessment of NPAs using SS-OCTA 24×20 mm. Several factors can affect OCTA image quality, including eyelid and eyelash interference, patient cooperation, the examiner’s imaging skills, and the presence of motion artifacts. Overcoming these challenges is essential for the future use of OCTA in the precise assessment of NPAs.

**Figure 6:**
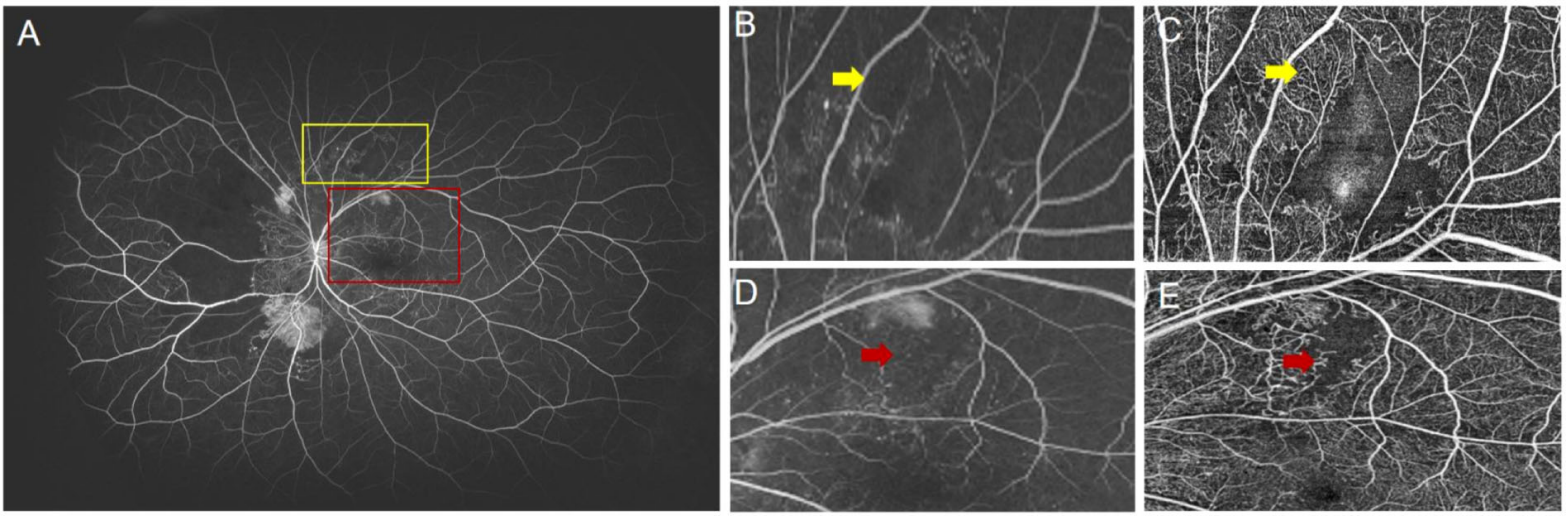
**(A)** UWFA image of a patient with proliferative diabetic retinopathy (PDR). The yellow box indicates the magnified region shown in **(B)**, and **(C)** shows the corresponding SS-OCTA image of the same area. The red box indicates the region shown in **(D)**, with **(E)** being the corresponding SS-OCTA image. **(B)** The yellow arrow indicates a region in the UWFA where it is difficult to determine the presence of blood perfusion. **(C)** The yellow arrow in the SS-OCTA image clearly shows blood perfusion in the same region. **(D)** The red arrow indicates an unclear NP boundary in the UWFA image. **(E)** The red arrow in the SS-OCTA image clearly defines the NP boundary.

Although OCTA’s current FOV limits its ability to detect NPAs in the retinal periphery, its superior detection within the scanned area is notable. In this study, UWFA was the gold standard for NPA detection, but some NPAs detected by SS-OCTA were difficult or impossible to identify on UWFA (**Figure 6**). This is likely due to SS-OCTA’s ability to visualize finer capillary details, providing clearer vascular boundaries, leading to higher inter-rater consistency (ICC = 0.979) compared to UWFA (ICC = 0.918). Previous studies, including those by LJ and Sawada et al. support these findings, highlighting SS-OCTA’s higher detection rate for NPAs (8, 15, 16). Despite manual annotation subjectivity, SS-OCTA’s resolution minimizes this issue. Its expanding FOV shows promise for future applications in NPA detection.

The ISI reflects the degree of retinal ischemia. Our study demonstrated a strong positive correlation between ISI measured by UWFA and SS-OCTA (ρ = 0.88,p<0.001), indicating that SS-OCTA, despite its smaller field of view, provides a reliable indication of retinal ischemia within the scanned area. Interestingly, the ISI was consistently higher with SS-OCTA compared to UWFA across all quadrants. A possible explanation for this discrepancy, as suggested by our heat map analysis, is that SS-OCTA’s 24×20 mm field of view may encompass regions with a higher density of NPAs, thus skewing the ISI toward higher values within the scanned area.

In clinical practice, based on the findings of our study, we recommend SS-OCTA for the initial screening of DR patients, especially for specific populations such as those with fluorescein allergies, pregnant women, and patients with renal impairment. SS-OCTA is also preferred for patients requiring frequent short-term follow-up. In contrast, for patients with severe NPDR or higher at the time of initial DR diagnosis, UWFA is more suitable for a comprehensive assessment of the disease. Additionally, if a detailed analysis of DR-related indicators is required, UWFA provides more accurate data for research purposes.

Our study has several limitations. First, as a retrospective, single-center study with a relatively small sample size, the generalizability of the findings may be limited, particularly due to the stringent image quality requirements for SS-OCTA. The incidence of artifacts in OCTA images is relatively high, leading to a higher exclusion rate when including images. The exclusion of these images may impact the reliability and generalizability of the results, which necessitates further research with a larger sample size to address this issue. The focus on NPAs, less common in patients with mild to moderate DR, led to an uneven distribution across DR stages, potentially impacting the comparative analysis. Additionally, important DR features such as NV and IRMAs were not included, limiting the scope of the study.

Despite these limitations, this study provides key insights into the comparative utility of SS-OCTA and UWFA for detecting NPAs in DR. Due to SS-OCTA’s limited field of view, it cannot fully replace UWFA, especially with significant loss of mid-peripheral NPAs. However, SS-OCTA remains a valuable tool for assessing retinal ischemia within its scanning range. Our findings also suggest NPAs are more frequent in the inferonasal quadrant, underscoring the importance of monitoring this region for early diagnosis and management of DR progression.

## Data Availability

The raw data supporting the conclusions of this article will be made available by the authors, without undue reservation.
