# Supplementary figures and images for: Comparison of widefield swept-source optical coherence tomography angiography and ultra-widefield fluorescein angiography in the detection of non-perfusion areas in diabetic retinopathy
Source: Front Endocrinol (Lausanne). 2025 Apr 8;16:1521837. doi: 10.3389/fendo.2025.1521837 (PMC12011578; doi:10.3389/fendo.2025.1521837)

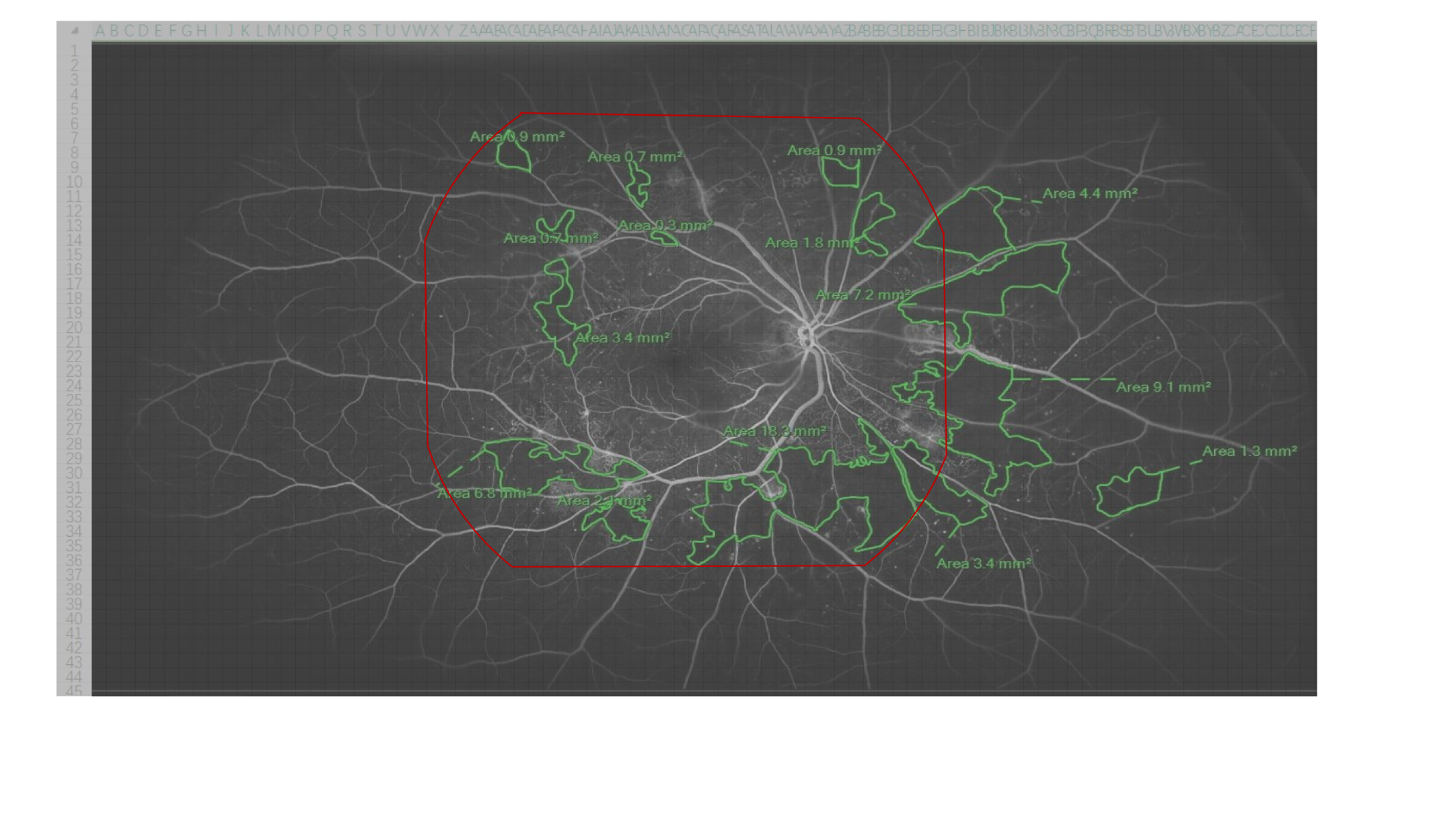

Supplement: Supplementary file 1 [file Image1.tiff]
