# Supplementary material for: Comparison of widefield swept-source optical coherence tomography angiography and ultra-widefield fluorescein angiography in the detection of non-perfusion areas in diabetic retinopathy
Source: Front Endocrinol (Lausanne). 2025 Apr 8;16:1521837. doi: 10.3389/fendo.2025.1521837 (PMC12011578; doi:10.3389/fendo.2025.1521837)
Supplement: Supplementary file 2 [file Table1.docx]

Supplementary Material

**Supplementary Figure 1.** This illustration shows the overlay of UWFA and SS-OCTA images aligned by the optic disc, macular center, and vascular patterns. All images were uniformly aligned to maintain consistency in coordinate calculations. The merged image is placed on a 45x85 semi-transparent grid, allowing the marked areas of non-perfusion (NP) to be clearly visible. In the grid, squares marked with NP are labeled as "1", and those without NP are labeled as "0", facilitating the observation of NP distribution across the image.
